# Identification of Internationally Disseminated Ceftriaxone-Resistant *Neisseria gonorrhoeae* Strain FC428, China

**DOI:** 10.3201/eid2507.190172

**Published:** 2019-07

**Authors:** Shao-Chun Chen, Yan Han, Liu-Feng Yuan, Xiao-Yu Zhu, Yue-Ping Yin

**Affiliations:** Chinese Academy of Medical Sciences and Peking Union Medical College, Nanjing, China (S.-C. Chen, Y. Han, X.-Y. Zhu, Y.-P. Yin);; Chinese Center for Disease Control and Prevention, Nanjing (S.-C. Chen, Y. Han, X-.Y. Zhu, Y.-P. Yin);; Beijing Ditan Hospital Capital Medical University, Beijing, China (L.-F. Yuan)

**Keywords:** Neisseria gonorrhoeae, antimicrobial resistance, internationally disseminated clone, bacteria, ceftriaxone resistance, sexually transmitted infections, China

## Abstract

In 2016, we identified a ceftriaxone-resistant *Neisseria gonorrhoeae* isolate in China. The strain genotype was identical to the resistant clone FC428 that originated in Japan. Enhanced international collaborative surveillance programs are crucial to track the transmission of the ceftriaxone-resistant clones.

Ceftriaxone has been used to treat gonorrhea in China and most other countries for >1 decade, but the level of decreased susceptibility or clinical resistance to ceftriaxone has increased (*1*). Moreover, the international spread of ceftriaxone-resistant clones has been recognized as a threat to effective control of gonorrhea (*2*). We describe an imported ceftriaxone-resistant *N. gonorrhoeae* strain isolated in China in 2016.

The patient was a heterosexual man in his late twenties. He reported unprotected 1-night heterosexual sex in Beijing in July 2016. Urethral discharge with dysuria occurred 3 days after the sexual activity. He was prescribed oral cephalosporin when he visited a private clinic in July. Because the urethral discharge did not resolve, he visited the sexually transmitted diseases clinic in Beijing Ditan Hospital (Beijing, China) in August.

Laboratory analysis of a urethral swab sample found gram-negative diplococci within leukocytes. Culture and nucleic acid amplification test were positive for *N. gonorrhoeae*. Screening for other sexually transmitted infections by nucleic acid amplification test was negative for *Chlamydia trachomatis*, *Ureaplasma urealyticum*, and *Mycoplasma hominis*.

The patient was treated with a 1-g intravenous dose of ceftriaxone once per day for 3 days. His symptoms improved after 3 days, and a test-of-cure by culture showed the treatment was successful. A telephone follow-up after 1 month indicated a lack of urethral discharge, and the patient provided information that his female sexual partner worked in a nightclub and had sexual contact with men from foreign countries.

The bacterial isolate was transferred to the reference laboratory at the National Center for Sexually Transmitted Disease Control, Chinese Center for Disease Control and Prevention (Nanjing, China). Gram staining and a carbohydrate utilization test confirmed *N. gonorrhoeae*. We confirmed antimicrobial susceptibilities to ceftriaxone, cefixime, spectinomycin, azithromycin, ciprofloxacin, and tetracycline for this isolate by using the agar dilution method. The strain was resistant to ceftriaxone (MIC 0.5 mg/L), cefixime (MIC 1 mg/L), tetracycline (4 mg/L), and ciprofloxacin (>32 mg/L) and susceptible to azithromycin (MIC 0.25 mg/L) and spectinomycin (MIC 16 mg/L) in accordance with the European Committee on Antimicrobial Susceptibility Testing protocol (http://www.eucast.org/clinical_breakpoints).

We performed *N. gonorrhoeae* multiantigen sequence typing (NG-MAST) (*3*) and multilocus sequence typing (MLST) (*4*) to identify the sequence types (STs). The MLST type was ST1903, and the NG-MAST type was ST3435. We used *N. gonorrhoeae* sequence typing for antimicrobial resistance (NG-STAR) (*5*) to identify the characteristics of resistance determinants. The NG-STAR type was ST233, which contains a type 60 mosaic *penA* allele (*penA* 60.001), −35A Del in the *mtrR* promoter (*mtrR*1), G120K-A121D in PorB (PorB8), L421P in PonA (PonA1), S91F-D95A in GyrA (GyrA7), S87R in ParC (ParC3), and wild-type 23srRNA (23 srRNA0).

The genotype (MLST1903/NG-MAST3435/NG-STAR233) of this isolate was identical to the 2 ceftriaxone-resistant *N. gonorrhoeae* (FC428 and FC460) isolated in 2015 in Japan (*6*) and similar to other resistant strains isolated in 2017 in Denmark (*7*), Canada (*8*), and Australia (*9*) (Table). Type 60 mosaic PenA (*penA* 60.001), which contained A311V and T483S alterations, was the key ceftriaxone resistance mutation and typical of this internationally disseminated resistant clone.

**Table:** Antimicrobial susceptibility and molecular characteristics of ceftriaxone-resistant *Neisseria gonorrhoeae*, China*

Strains	Year	Country (reference)	Sexual contact history	MIC, mg/L						MLST	*porB*	*tbpB*	NG-MAST	*penA*	NG-STAR
				TET	SPT	CRO	CIP	AZI	CEF						
Japan-FC428	2015	Japan (*6*)	NA	0.5	8	0.5	>32	0.25	1	1903	1053	21	3435	60	233
Japan-FC460	2015	Japan (*6*)	NA	0.5	8	0.5	>32	0.25	1	1903	1053	21	3435	60	233
China-BJ16148	2016 Aug	China (this study)	China	4	16	0.5	>32	0.25	1	1903	1053	21	3435	60	233
Denmark-GK124	2017 Jan	Denmark (*7*)	Denmark, China, Australia	NA	8	0.5	>32	0.5	1	1903	1053	33	1614	60	233
Canada-47707	2017 Jan	Canada (*8*)	China, Thailand	4	16	1	>32	0.5	2	1903	1053	33	1614	60	233
Australia-A7846	2017 Apr	Australia (*9*)	Cambodia, Philippines	2	8	0.5	>32	0.25	NA	1903	1053	33	1614	60	233
Australia-A7536	2017 Aug	Australia (*9*)	China	4	8	0.5	>32	0.25	NA	1903	9300	21	15925	60	233

*AZI, azithromycin; CEF, cefixime; CIP, ciprofloxacin; CRO, ceftriaxone; MLST, multilocus sequence typing; NA, not available; NG-MAST, *N. gonorrhoeae* multiantigen sequence typing; NG-STAR, *N. gonorrhoeae* sequence typing for antimicrobial resistance; SPT, spectinomycin; TET, tetracycline.

The timeline and epidemiologic data of all previous reports of the infections suggest this clone originated in Japan in 2015 and was disseminated to China, Denmark, Canada, and Australia afterward. Moreover, this resistant clone may have a fitness advantage over previously reported “superbug” H041 and has successfully spread worldwide (*9*). Accordingly, enhancing international collaborative surveillance on the ceftriaxone-resistant clone is crucial.

In conclusion, we identified a ceftriaxone-resistant *N. gonorrhoeae* strain that has sustainably transmitted in several countries for ≈3 years. These findings indicate an imported risk and a further transmission of resistant clones in China and demonstrate the need for enhanced local and global gonococcal antimicrobial surveillance to track the emergence and dissemination of resistant strains for timely control of spread (*10*).
